# Design of the OverCool study: Lung-conservative liquid ventilation for the induction of Ultra-Rapid Cooling after Cardiac Arrest (OverCool)

**DOI:** 10.1016/j.resplu.2025.100926

**Published:** 2025-03-10

**Authors:** Renaud Tissier, Fabio Silvio Taccone, Lionel Lamhaut, Eric Vicaut, Fabrice Paublant, Jean-Damien Ricard, Alain Mercat, Alain Cariou

**Affiliations:** a Paris Est Créteil, INSERM, IMRB F-94010 Créteil, France; b Ecole Nationale Vétérinaire d’Alfort, IMRB, AfterROSC Network, F-94700 Maisons-Alfort, France; c Orixha, F-94700 Maisons-Alfort, France; d Department of Intensive Care, Hôpital Universitaire de Bruxelles (HUB), Université Libre de Bruxelles (ULB), Brussels, Belgium; e SAMU de Paris-ICU, Necker University Hospital, Assistance Publique-Hôpitaux de Paris, Université Paris-Cité, F-75015 Paris, France; f Clinical Trial Unit, Groupe Hospitalier Lariboisière - Fernand-Widal, APHP, Université Paris Cité, Paris, France; g Université Paris Cité, UMR1137, Inserm, APHP, Hôpital Louis Mourier, DMU ESPRIT, Service de Médecine Intensive, Colombes, France; h CHU Angers, Angers, France; i Service de Médecine Intensive et Réanimation, Hôpital Cochin, APHP, Université Paris Cité, Paris, France

**Keywords:** Cardiac arrest, Resuscitation, Hypothermia, Target temperature management, Liquid ventilation, Neuroprotection

## Abstract

**Background:**

The therapeutic window within which induced hypothermia might be effective after cardiac arrest is still unknown. In animal cardiac arrest models, early induction and faster cooling are independently associated favorable outcome. However, induction of Ultra-Rapid Therapeutic Hypothermia (achieving core body temperature of 33.0 ± 0.5 °C within 60 min after the start of the procedure) still need to be evaluated in the human setting. Total liquid ventilation with temperature-controlled breathable liquids provided such rapid cooling (i.e. >15 °C/h cooling rate) in both small and large animals. This method was shown to improve neurological outcome in animals. A new medical device system, Vent2Cool, was developed for clinical use in the Intensive Care Unit to achieve ultra-rapid therapeutic hypothermia by total liquid ventilation to patients.

**Materials and methods:**

The non-blinded and single-arm OverCool pilot trial will evaluate the feasibility, cooling performance and safety of ultra-rapid therapeutic hypothermia by total liquid ventilation in 24 patients resuscitated after in- or out-of-hospital cardiac arrest. Inclusion criteria will include presumption to start ultra-rapid cooling procedure in the Intensive Care Unit within less than 120 min after resuscitation. The primary outcome will be the achievement of a core temperature of 33.0 ± 0.5 °C, as well as successful return to conventional gas ventilation within <60 min after procedure initiation. Secondary outcomes will include time to reach target temperature, vital status, systemic and pulmonary parameters and modified-Rankin Score at 28 days post- cardiac arrest.

**Conclusion:**

The OverCool study is a pilot study to validate performance and safety of ultra-rapid therapeutic hypothermia using total liquid ventilation for resuscitated cardiac arrest patients.

**Registration and authorization:**

NCT06798818. Authorized by the French “Agence nationale de sécurité du médicament et des produits de santé” and “Comité de Protection des Personnes” (Ethics Committee).

## Introduction

“Targeted Temperature Management (TTM)” within the hypothermic range of 32–34 °C, commonly referred as therapeutic hypothermia, has been proposed as an intervention to attenuate post-cardiac arrest syndrome (PCAS) in patients successfully resuscitated following out-of-hospital cardiac arrest (OHCA).^1^, ^2^ Following promising pilot studies in the early 2000 s, large-scale clinical trials have demonstrated that TTM at 33 °C with existing techniques does not significantly improve outcomes compared to sub-normothermia (36 °C)^3^ or strict normothermia (37 °C)^4^ after resuscitation. Current guidelines recommend to provide controlled normothermia in CA survivors,^5^ while whether therapeutic hypothermia might still benefit for some subgroup of patients remains unknown.^5^, ^6^

In contrast to the findings of most clinical trials, experimental studies frequently demonstrate that therapeutic hypothermia significantly improves neurological outcomes in animal models of cardiac arrest.^7^, ^8^ A recent meta-analysis of animal studies highlighted that the neurological benefits of hypothermia were independently associated with “faster cooling rates, lower target temperature of TTM within the range of 32–36 °C, and shorter duration of cooling”.^9^ Additionally, a recent Cochrane review, based on subgroup analyses, found that the beneficial effects of hypothermia were most pronounced when cooling was initiated within two hours of the return of spontaneous circulation (ROSC).^10^

A novel approach for achieving therapeutic hypothermia much faster than with traditional external or endo-vascular cooling systems is to perform total liquid ventilation (TLV) with cold breathable perfluorocarbon liquids, thereby using the lungs as thermal exchangers. This innovative technique has been shown in preclinical studies to rapidly lower core body temperature within minutes, providing significant neurological benefits across multiple animal models^11^, ^12^, ^13^, ^14^, ^15^, ^16^ while preserving normal gas exchange due to the unique properties of these breathable liquids. The method is engineered to achieve a target temperature of 33 °C in body core within one hour of initiation, irrespective of initial patient temperature. This is accomplished through a lung-conservative TLV strategy that precisely controls liquid pressure and maintains lung liquid volume below the functional residual capacity.

Here, we describe the design of a “first-in-human”, open-label, single-arm, non-comparative pilot clinical study, named **“***Lung-Conservative Liquid Ventilation for the Induction of Ultra-Rapid Cooling After Cardiac Arrest*” (OverCool), aiming at evaluating the performance and safety of ultra-rapid therapeutic hypothermia (URTH) induction by TLV.

## Methods

OverCool is an open-label, two-center, pilot study prospectively registered as NCT06798818 and authorized by the French authorities (Agence nationale de sécurité du médicament et des produits de santé) and Ethics Committee (Comité de Protection des Personnes). The trial is planned to recruit across Cochin Hospital (Assistance-Publique Hôpitaux de Paris, Paris, France) and Angers Hospital (CHU Angers, Angers, France). The protocol was developed by the trial investigators in accordance with national legislation, Good Clinical Practice and declaration of Helsinki. The trial is sponsored by ORIXHA, developer of the Vent2Cool system, and supervised and monitored by a Contract Research Organization (Clinact- MultiHealth, Velizy, France). The protocol manuscript was written in concordance with the SPIRIT guidelines. Additional information is provided in the Supplementary material.

### Trial objectives

The primary objective is to demonstrate the ability of the Vent2Cool medical system (Orixha, Paris, France) to perform hypothermic TLV. Vent2Cool performance and efficacy have been rigorously validated *in vitro* and *in vivo*.^11^, ^12^, ^13^, ^14^, ^15^, ^16^ URTH targeted the achievement of core body temperature of 33.0 ± 0.5 °C within 60 min after the start of the procedure in patients resuscitated after intra- or extra-hospital cardiac arrest.

As secondary objectives, the study will aim at demonstrating: the safety of the procedure, the cooling performance, the ability to be implemented in the emergency setting of post-cardiac arrest management, clinical outcomes and impact on biological markers.

### Primary and secondary endpoints

The primary endpoint is the success of the procedure, defined as the ability to reach a body core temperature (bladder recommended) of 33.0 ± 0.5 °C and a safe return to conventional mechanical ventilation within less than one hour after Vent2Cool start. Start of the Vent2Cool procedure is defined as the opening of the Vent2Cool patient connector valve. Secondary endpoints are shown in Table 1, Table 2.

**Table 1:** Summary of the secondary safety endpoints.

**Secondary safety endpoints**
• SaO_2_, PaO_2_ and PaCO_2_ at inclusion, 10 and 25 min after the start of Vent2Cool procedure and 1 h, 2 h, 12 h, 24 h, 48 h and 72 h after the end of procedure
• Occurrence of an episode of severe oxygen desaturation (SpO_2_ < 75%) on pulse oximeter lasting at least 5′ during the Vent2Cool procedure
• Occurrence of severe hypercapnia (PaCO_2_ > 75 mmHg); hyperoxemia (PaO_2_ > 400 mmHg); hypoxemia with PaO_2_ < 75 mmHg in arterial blood at t = 10 and 25 min after starting Vent2Cool procedure
• Occurrence of an episode of major systemic hypotension (invasive or non-invasive measurement) with systolic blood pressure < 60 mmHg during Vent2Cool procedure
• Heart rate measured from the EKG or blood pressure signal at 10 and 25 min after starting Vent2Cool procedure and at t = 1 h, 2 h, 12 h, 24 h, 48 h and 72 h after the procedure
• Systemic systolic and diastolic blood pressure at 10 and 25 min after starting Vent2Cool procedure and at t = 1 h, 2 h, 12 h, 24 h, 48 h and 72 h after the procedure
• Presence of arrhythmia or unexpected electrocardiographic abnormality
• Early termination of the Vent2Cool procedure before reaching a core target temperature below 33 ± 0.5 °C (failure of cooling induction)
• Number and percentage of patients for whom the core temperature has reached a value lower than 30 or 32 °C within 60 min after the start of the Vent2Cool procedure
• Adverse events related to Vent2Cool procedure, i.e., appearance of a perfluorothorax, unexpected cardiac arrhythmia or major hemodynamic instability, major hypoxemia during or immediately after the Vent2Cool procedure (i.e., during the first hour following the end of the procedure)
• Ventilatory parameters after the Vent2Cool procedure, i.e., measured values of positive end-expiratory pressure (PEEP), end-inspiratory pause pressure (Plateau pressure), driving pressure and static pulmonary compliance at t = 1 h, 2 h, 12 h, 24 h, 48 h and 72 h after the Vent2Cool procedure
• Occurrence of pulmonary infection in the ICU
• Presence or absence of pleural collection of perfluorocarbon in the lungs on thoracic computed tomography examination in patients surviving on Day 28 after cardiac arrest.

**Table 2:** Summary of the secondary endpoints for cooling performance, implementation in the care pathway, survival and neurological outcome, and clinical pathology.

**Secondary endpoint of the demonstration of cooling performance**
• Body core (bladder recommended) temperature at start of Vent2Cool procedure and every 1 min for 60 min thereafter. Then, every 15 min for an additional hour and every 2 h until 36 h after the end of Vent2Cool procedure.
• Cooling Rate in body core temperature, calculated as follows: temperature at start of Vent2Cool procedure – 33.5 °C / duration to achieve a core temperature of 33.5 °C
• Delay between the start of the Vent2Cool procedure and body core temperature of 33.5 °C
• Actual duration of the Vent2Cool procedure (from the start of the procedure on the patient to the end of the procedure)
• Delay between ROSC and body core temperature of 33.5 °C.
• Vent2Cool performance parameters based on device data recorded during the Vent2Cool procedure
**Secondary endpoints related to Vent2Cool implementation in the care pathway**
• Time of start of Vent2Cool procedure from the time of return of spontaneous circulation (ROSC).
• Time of start of Vent2Cool procedure from the patient's admission to ICU department in which it was implemented.
**Secondary criteria of survival and neurological outcome after cardiac arrest**
• Vital status after ICU discharge and/or hospital discharge (if before Day 28) and systematically at Day 28 after inclusion. In case of death, cause of death.
• modified Rankin Scale (mRS) at Day 28 after inclusion.
• Glasgow score (Glasgow coma scale) at inclusion, days 1, 2, 3 and 28 after inclusion
• SOFA score at Day 1 and 3
• Glasgow outcome score at day 28 after inclusion
• Pupillometry (presence of pupillary light reflex and Neurological pupil index (NPi)) at Day 1, 2 and 3 after cardiac arrest
• Value of the modified Cardiac Arrest Hospital Prognosis (mCAHP) Score. Scores of either < 80; [80; 105[ or > 105 predict of risk of poor outcomes of respectively at least 40%, at least 80% or at least 95%.
• Number of patients for which the mCAHP score predicted a poor outcome with a risk of at least 40% (mCAHP < 80), at least 80% (mCAHP [80; 105[) or at least 95% (mCAHP ≥ 105) vs. the total number of patients within each mRS category at Day 28.
• For patients discharged alive from hospital and / or alive at Day 28: number of days under mechanical ventilation, number of days in intensive care unit, number of days before hospital discharge, if applicable
**Secondary endpoints for clinical pathology**
• Urea, creatinine, ASAT, ALAT, γ-GT, blood count at inclusion, day 1 and day 3 after the Vent2Cool procedure
• Neurofilament light chain (NFL) and Neuron Specific Enolase (NSE) at inclusion, day 1 and day 3 after the Vent2Cool procedure

### Eligibility criteria

Eligible patients will be unconscious patients resuscitated after extra- or intra-hospital cardiac arrest and admitted to the Intensive Care Units (ICU). Cardiac arrest from presumed cardiac or asphyxial origin, regardless of the heart rate initially recorded, will be included. Patients must be aged between 18 and 84 years with an ideal body weight (IBW) comprised between 40 and 93 kgs. Inclusion and exclusion criteria are shown in Table 3.

**Table 3:** Summary of inclusion and exclusion criteria.

Inclusion criteria	Exclusion criteria
• Patient resuscitated after extra- or intra-hospital cardiac arrest, from presumed cardiac or asphyxial origin, regardless of the heart rate initially recorded.	• Conscious patient
• Age between 18 and 84 years old	• Patient Ideal Body Weight less than 40 kgs or more than 93 kgs
• Time between collapse and return of spontaneous circulation (ROSC) < 60 min	• Traumatic cardiac arrest, drowning, exsanguination or sepsis
• Presumption, at the time of inclusion, of a possibility of starting Vent2Cool within a period of less than 120 min after ROSC.	• Temperature at admission < 34.0 °C
• Unconscious (GCS < 8, not able to obey verbal commands after sustained ROSC)	• Need for veno-arterial extracorporeal circulation before ROSC
• Patient affiliated with a social security system (If applicable)	• One of the following signs at echocardiography at hospital admission: - Acute core pulmonale, defined by right ventricular enlargement with interventricular septal deviation, associated with a Tricuspid Annular Plane Systolic Excursion (TAPSE) < 12 mm and need for vasopressor agents - Time-velocity integral of subaortic flow < 10 m/s
• Consent of the member of patients’ family or that of the trusted person if present and able to understand; otherwise certificate of emergency inclusion in compliance with applicable regulations.	
• Vent2Cool must be connected to a standard cuffed ETT of 7.0 mm to 9.0 mm internal diameter for the procedure. For 7.0 mm ETT, the IBW setting should not exceed 75 kg. For ETTs of 7.5 to 9.0 mm, the full range of the IBW setting (i.e. 40 to 93 kg) can be applied.	
	• Wrong positioning or damage of the endotracheal tube.
	• Women under 50 years old (childbearing age) or for women of > 50 years old, a positive pregnancy test (urinary) in case of pregnancy suspicion by the investigator. Possible breastfeeding during the study.
	• Suspicion of intracranial bleeding
	• History of severe Chronic Obstructive Pulmonary Disease (COPD) with long-term home oxygen therapy
	• Acute respiratory pathology (pneumothorax, pleurisy, pneumonia, suspicion of contusion or intra-pulmonary hemorrhage following resuscitation)
	• COVID-19 positive test in case of clinical suspicion and/or epidemic context
	• Excessive mucus in the upper airways
	• Patient presenting at least one of the following criteria in the mechanical ventilation parameters at inclusion: PEEP > 8 cm H_2_O, Plateau pressure > 25 cm H_2_O, Driving pressure > 15 cm H_2_O
	• Patient with at least one of the following criteria at inclusion: PaO_2_ < 80 mmHg, PaO_2_/FiO_2_ < 150 mmHg, PaCO_2_ > 48 mmHg

### Consent, data collection and follow-up

In the context of comatose patients resuscitated after cardiac arrest, it will not be possible to obtain prior informed consent from the patient himself. An emergency inclusion procedure will then be made possible. The detailed process of the inclusion process is provided in Supplemental Table 1.

### Study intervention and target temperature management

**Fig. 1:**
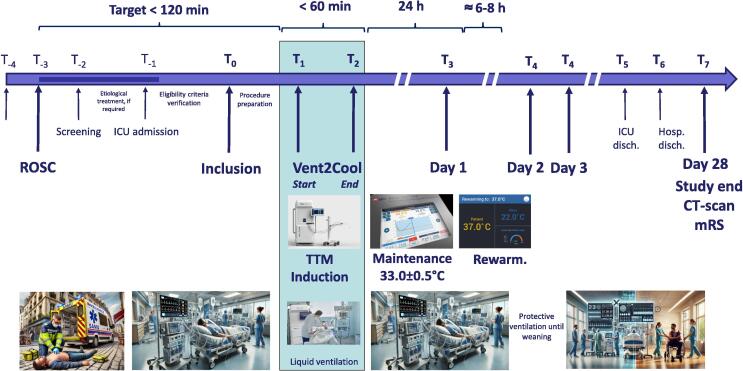
Schematic representation of the study design. *ROSC, resumption of spontaneous circulation; TTM, target temperature management; mRS, modified Rankin Score.*

Overall study design is illustrated by Fig. 1. After admission to the ICU and putative etiological treatment, if required, eligible patients could be included for URTH induction by Vent2Cool. Patients will be sedated and paralyzed with atracuronium and monitoring of muscle paralysis is recommended whenever possible. The maintenance of sedation and muscle paralysis after the Ven2Cool procedure will be left to the discretion of the physicians. Surface cooling device, that will be necessary to maintain hypothermia after induction by Ven2Cool, will be pre-positioned in order to avoid mobilization immediately after this procedure. However, the surface cooling system will not be active at this stage and until the end of the Vent2Cool procedure.

**Fig. 2:**
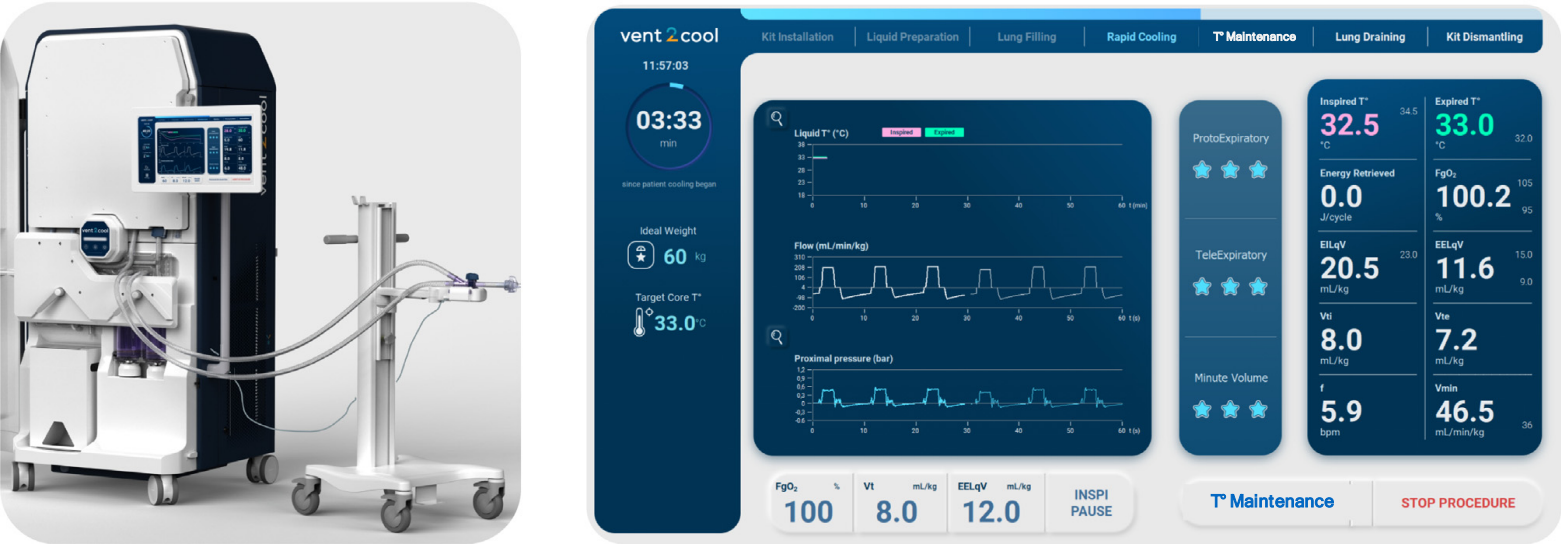
Schematic illustration of the Vent2Cool system (left panel) and its graphic-user interface (right panel) delivering total liquid ventilation and ultra-rapid hypothermia.

**Fig. 3:**
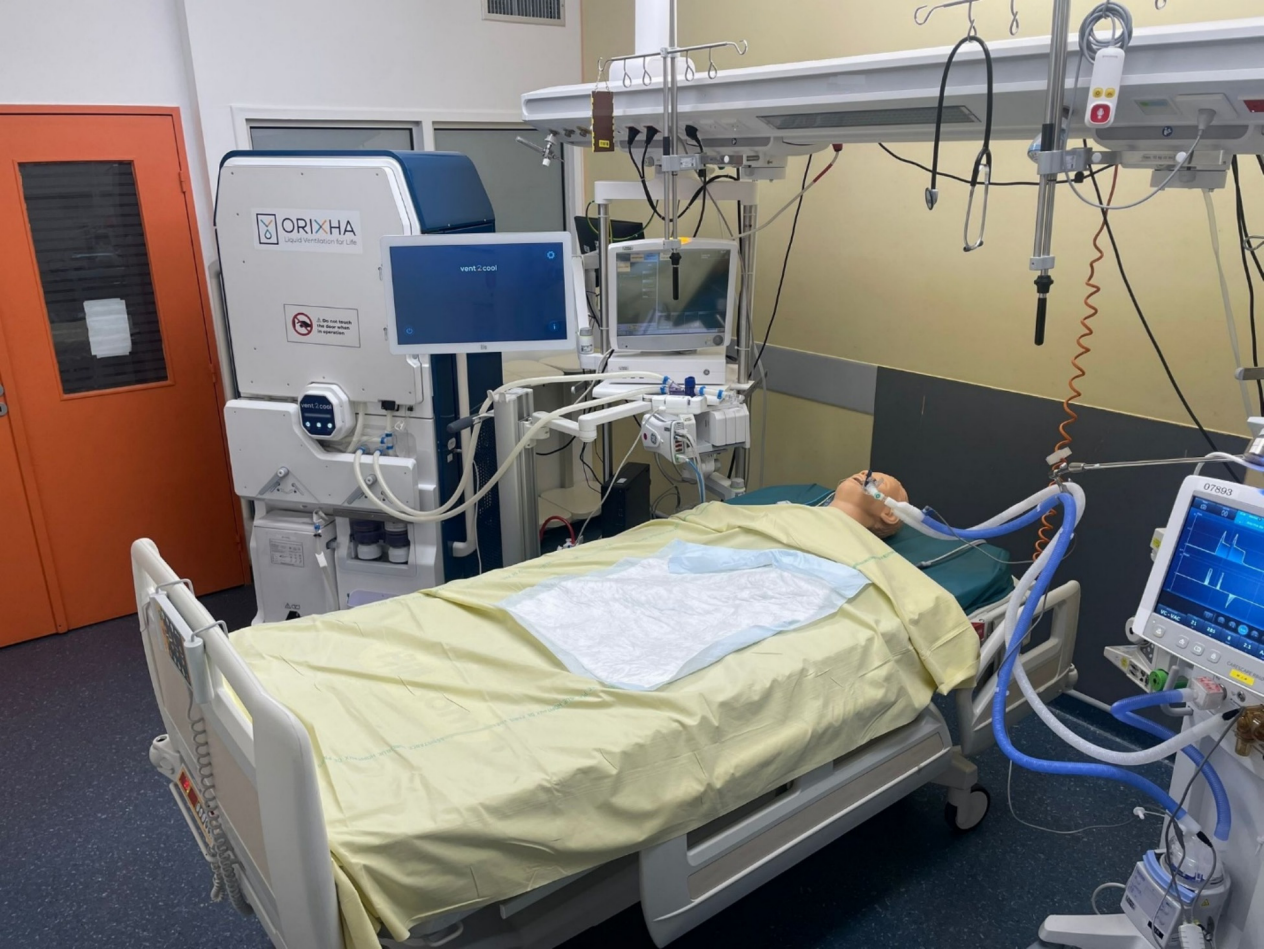
Picture of the Vent2Cool system in the Intensive Care Unit.

Once the inclusion process has been completed, the Vent2Cool system will be set up according to instruction for use, after installation of a single-use Breathing Circuit as well as a single-use bag containing sterile breathable liquid called “Liquid2Breathe”. Schematic illustrations and picture of the Vent2Cool system are illustrated in Fig. 2, Fig. 3, respectively. Prior to the beginning of the Vent2Cool procedure, the user will enter the Ideal Body Weight of the patient and confirm the pre-set parameters for end-expiratory liquid volume (EELqV), liquid tidal volume (Vt) and Fraction of oxygen in the gas mixture (FgO_2_). This is performed on the Vent2Cool touch screen in order to personalize the treatment (i.e. quantity of breathable liquid) for the given patient. The user will start the Vent2Cool procedure after disconnection of the endotracheal tube from the gas ventilator followed by a rapid secured connection to the Vent2Cool connector. Upon switching the Vent2Cool valve ON, the Vent2Cool system automatically fills the lungs with breathable liquid and starts URTH induction. Throughout the procedure, the user can modify EELqV (10–20 mg/kg IBW), Vt (6–9 ml/kg IBW) and the FgO_2_ (50–100%). Of note, FgO_2_ is fully distinct from the inhaled fraction of oxygen used in conventional ventilation as this is the fraction of oxygen in the gas mixture delivered by Vent2Cool into the breathable liquid, but not the fraction of in in the breathable liquid. The end of the procedure is expected to occur after reaching a core temperature of 33.0 °C and a procedure duration of less than 60 min. The investigator can stop the procedure at any moment for any safety reason. When stopping the procedure, the Vent2Cool system automatically withdraws the maximal amount of breathable liquids from the lung through a lung draining procedure. The user will end the Vent2Cool procedure by closing the patient connector valve a reconnecting the endotracheal tube to the gas ventilator. If required, the investigator will use bronchoscopy to suction residual amounts of breathable liquids. It will be suggested to maintain a Vt less than or equal to 6 ml/kg and a positive end-expiratory pressure (PEEP) of at least 5 cmH_2_0 for a minimum of 8 h following the procedure. The use of a heat and moisture exchanger (HME) on the respiratory circuit will be discouraged during this same period and the use of a heated humidifier should be favored.

After resumption of conventional ventilation, the body temperature of the patients will be maintained at 33.0 ± 0.5 °C by surface cooling devices for 24 h. As described above, the “pads” set up initially will be connected to the external thermal control device. After 24 h of hypothermia, patients will be rewarmed according to current protocols in the investigator's site. A rewarming rate of 0.25 to 0.5 °C/h will be recommended.

### Patients’ follow-up

After rewarming, patients will be followed according to the hospital practices. They could be weaned from the ventilator and extubated, according to the centers procedure with, e.g., PaO_2_ (arterial oxygen tension) / FiO_2_ (Inhaled oxygen) ratio > 200 mmHg, FiO_2_ < 50%, PEEP < 8 cmH_2_O, lack of vasopressor administration, cough reflex and respiratory rate / tidal ratio < 105. The previously mentioned secondary end-points will be monitored until Day 3 after cardiac arrest, as shown in Table 1, Table 2. We will collect the final cause of the initial of cardiac arrest, coronarography data (if available), results of other etiological examinations (e.g., brain CT-scan, chest CT-scan), electroencephalogram, time of rewarming and time of normothermia achievement.

Study will end by a last visit by the investigator 28 days after cardiac arrest (“face to face”). During this visit, the investigator will record the number of days under mechanical ventilation, the occurrence or not of weaning from mechanical ventilation, the number of days in intensive care unit (before transfer to another ward), the number of days before hospital discharge, the number of days alive and permanently out of hospital, in case of death, cause of death, the current treatments and dosages, body core temperature any delayed adverse effects that can be attributable to Vent2Cool. A chest-CT scan will be performed to evaluate the presence or nor pleural collection of PFOB.

### Study schedule

The enrolment phase in expected to last 11 months. Since study duration is 28 days for each patient, the overall study is expected to last 12 months.

### Sample size and statistical analysis

The analyses will be conducted on the following patient sets: Safety population (SAF), Full Analysis Set (FAS) and Population Per Protocol (PP). The SAF includes all included and patients treated by Vent2Cool. The FAS (according to the Intent-to-Treat (ITT) principle) includes all treated patients, who had one post-baseline evaluation. The PP population includes all patients of the FAS except those with any major protocol deviation, that might significantly affect the completeness, accuracy, and/or reliability of the study data. The main endpoint analysis and secondary endpoint analyses will be based on the Full Analysis Set. A supportive analysis will be based on the Per Protocol population if more than 10% of patients are excluded from FAS.

A summary table and a flow chart will be presented for each patient population presenting the number of patients at each assessment and identifying the number of patients who withdrew over time, with the reason of premature study termination. Patients may withdraw from the study at any time, with or without reason and without prejudice to further treatment. Data that have already been collected on prematurely withdrawn patients will be retained and used for analysis, but no new data will be collected after withdrawal. Patients lost to follow-up, after hospital discharge before the end of the study, will be replaced. Data collected until the last available visit of these patients will be analyzed.

Based on an estimated success rate of around 80%, a sample of 24 analyzable patients was calculated as required to achieve sufficient precision of the success rate using Wilson's score interval, with a 95% confidence interval. Specifically, 19 of the 24 patients will have to undergo the procedure successfully, giving an expected success rate of 79.2% (95% CI [59.5%; 90.8%]).

Quantitative parameters will be described using the following summary descriptive statistics: number of patients, mean, standard deviation, median, first and third quartiles, and minimum and maximum values. Mean and standard deviation values will be reported to one decimal place greater than the data were collected. Median, first and third quartiles, minimum and maximum values will be reported with the same precision, as they were collected. Qualitative parameters will be described using frequencies and percentages.

## Discussion

The Overcool study will be the first study investigating URTH and TLV in resuscitated patients after cardiac arrest. It takes place in the context of three major ongoing studies on TTM and hypothermia. First, the “*Sedation, Temperature and Pressure After Cardiac Arrest and Resuscitation*” (STEPCARE, NCT05564754) study will include three different interventions focusing on sedation targets, mean arterial pressure targets and temperature targets. It will compare the effect of fever management with or without a feedback-controlled device in 3500 patients, with an estimated completion date in June 2026. Second, the “*Influence of Cooling Duration on Efficacy in Cardiac Arrest Patients*” study (ICECAP, NCT04217551) will enroll 1800 patients in order to determine whether longer durations of cooling may improve the neurological recovery in resuscitated patients. The ICECAP study is response-adaptive and “duration-finding” with an expected completion date in August 2028. Finally, the “*Prehospital Resuscitation Intranasal Cooling Effectiveness Survival Study 2*“ (PRINCESS2, NCT06025123) study will evaluate the impact of ultra-early trans-nasal evaporative cooling after cardiac arrest on survival with complete neurologic recovery as compared to currently recommended normothermia.^17^ It will enroll 1022 patients after out-of-hospital cardiac arrest with initial shockable rhythm. It is planned to be completed by May 2028. The OverCool study, which aims to be completed in 2026, will provide additional information with a novel and more potent cooling approach for URTH. If the cooling performance of Vent2Cool is confirmed for URTH induction, it could become a relevant approach for the investigation of the therapeutic window of hypothermic TTM, together with pre-existing techniques for good-quality TTM.

Beyond the evaluation of the performance of URTH by TLV, the OverCool trial will be the first study to properly investigate TLV in humans with the first specifically designed ventilator, the Vent2Cool liquid ventilator. In 1989, Greenspan et al. pioneered the use of liquid ventilation in a neonate experiencing severe respiratory failure unresponsive to conventional ventilation.^18^ The intervention involved two 3-minute periods of liquid ventilation, separated by 15 min. During the procedure, pre-oxygenated perfluorochemicals were introduced via a burette, retained in the lungs for 15 s, and then drained under gravity. The following year, a case series involving three neonates, including this first one, demonstrated improvements in lung compliance in all three cases, with two showing enhanced oxygenation.^19^ Despite all the neonates succumbing within 19 h, the outcomes were attributed to the severity of their conditions rather than the failure of liquid ventilation, highlighting its potential clinical utility. Since these first clinical experiences, TLV was not further evaluated in patients as it required a dedicated device that was not available until now.^20^

A hybrid method was then proposed through lung filling with the perflubron as breathable liquid followed by superimposing gas tidal volumes, known as partial liquid ventilation (PLV). Hirschl et al. evaluated this PLV method in 19 patients, including adults, children, and neonates, all of whom were also treated by extracorporeal membrane of oxygenation (ECMO) as a backup for gas exchange. Improvements in alveolar-arterial oxygen gradients and static lung compliance were documented during PLV under these conditions.^21^ A multicenter trial then examined the safety and effectiveness of PLV in about 300 patients using two different targets for lung filling, corresponding to approximately 10 or 20 ml/kg.^22^ The study showed no improvement in ventilator-free days or 28-day all-cause mortality and increased risk of pneumothorax with PLV, halting further research for presumed safety concerns. The risk of pneumothorax was known prior to this study^21^ and linked to the risk of overdistension when gas entered lung areas not fully filled with liquid, highlighting the need for pressure-controlled ventilation. Unfortunately, this PLV pivotal inadequately utilized volume-controlled gas ventilation with tidal volumes averaging 9 mL/kg, which is considered harmful even in conventional ventilation. In addition, the study’s design required fluctuating PEEP for regular lung refilling to compensate for evaporative loss with no possibility to control actual lung liquid volume. This could easily explain the failure of PLV in this study. Accordingly, alternative approaches like TLV may provide the expected benefits of liquid ventilation in a safer manner^20^, ^23^. For instance, the “lung-conservative liquid ventilation” approach was proposed for short-term TLV and URTH induction, through lung liquid volume reliably maintained below functional residual capacity. If the OverCool trial confirms TLV safety after cardiac arrest, it could reignite interest in liquid ventilation as a supplemental or alternative treatment for respiratory distress, advancing care beyond current gas ventilation practices.

## Conclusions

The OverCool trial aims at demonstrating the performance and safety of URTH by TLV in resuscitated patients after cardiac arrest with the Vent2Cool system. Vent2Cool can be an innovative efficient approach to induce therapeutic hypothermia within 3 h after cardiac arrest. Its benefits on cardiac arrest clinical outcomes will need to be further investigated in comparative clinical studies. The OverCool trial can also pave the way for other applications of TLV in the ICU.

## CRediT authorship contribution statement

**Renaud Tissier:** Writing – review & editing, Writing – original draft, Supervision, Resources, Project administration, Methodology, Funding acquisition, Conceptualization. **Fabio Silvio Taccone:** Writing – review & editing, Methodology, Conceptualization. **Lionel Lamhaut:** Writing – review & editing, Methodology, Conceptualization. **Eric Vicaut:** Writing – review & editing, Methodology, Conceptualization. **Fabrice Paublant:** Writing – review & editing, Writing – original draft, Resources, Project administration, Methodology, Funding acquisition, Conceptualization. **Jean-Damien Ricard:** Writing – review & editing, Methodology, Conceptualization. **Alain Mercat:** Writing – review & editing, Supervision, Resources, Project administration, Methodology, Conceptualization. **Alain Cariou:** Writing – review & editing, Supervision, Resources, Project administration, Methodology, Conceptualization.

## Funding

The Overcool study is funded by Orixha.

## Declaration of competing interest

The authors declare that they have no known competing financial interests or personal relationships that could have appeared to influence the work reported in this paper.
