# Supplementary material for: Design of the OverCool study: Lung-conservative liquid ventilation for the induction of Ultra-Rapid Cooling after Cardiac Arrest (OverCool)
Source: Resusc Plus. 2025 Mar 10;23:100926. doi: 10.1016/j.resplu.2025.100926 (PMC11979425; doi:10.1016/j.resplu.2025.100926)
Supplement: Supplementary Data 1 [file mmc1.doc]

**Supplemental Table 1:** Additional information regarding the UltraCool trial, in compliance with the “Standard Protocol Items: Recommendations for Interventional Trials” (SPIRIT) recommendations (https://spirit-statement.org/). Numbers in brackets correspond to the item numbers in these recommendations.

| Section/item | Description | |
| --- | --- | --- |
| **Administrative information** | | |
| Title (1) | The OverCool Study: lung-cOnservative liquid VEntilation for the induction of ultra-Rapid COOLing after cardiac arrest (OverCool) | |
| Trial registration (2) | 2023-A02030-45 - N°EUDAMED: CIV-24-02-046114  Clinical trial.gov: NCT06798818 | |
| Protocol version (3) | 2.3 - 16/12/2024 | |
| Funding (4) | ORIXHA  Société par Actions Simplifiée, registered by RCS Lyon ‭838982858‬‬‬‬ - 4 hameau du Moulin d’Arche, 69450 Saint Cyr au Mont d’Or, France | |
| **Roles and responsibilities (5a to 5d)** | **Coordinating investigator:** Alain Cariou - Université Paris Cité, Hôpital Cochin (Paris, France)  **Scientific supervisor:** Renaud Tissier – Full Professor Ecole nationale vétérinaire d’Alfort and Inserm (Maisons-Alfort, France) ; Medical advisor at Orixha (Lyon, France)  **Steering committee (also called scientific committee):**  *Alain Cariou - Université Paris Cité, Hôpital Cochin (Paris, France)*  *Lionel Lamhaut - Université Paris Cité, SAMU de Paris (Paris, France)*  *Alain Mercat - CHU Angers ( Angers, France)*  *Fabrice Paublant - censor - Orixha (Lyon, France)*  *Jean-Damien Ricard - Université Paris Cité, Hôpital Louis Mourier (Colombes, France)*  *Fabio Taccone - Hôpital Erasme (Brussels, Belgium)*  *Renaud Tissier – Ecole nationale vétérinaire d’Alfort and Inserm (Maisons-Alfort, France) and Orixha (Lyon, France)*  *Eric Vicaut - Université Paris Cité, Unité de Recherche Clinique (URC) Saint-Louis Lariboisière Fernand-Widal (Paris, France*)  **Role of steering committee:** study design, data analysis, interpretation, writing of the report.  **Sponsor:** ORIXHA, Société par Actions Simplifiée, registered by RCS Lyon ‭838982858‬‬‬‬ - 4 hameau du Moulin d’Arche, 69450 Saint Cyr au Mont d’Or, France  **Role of study sponsor:** involved in the above mentioned activities through representatives in the steering committee, with an ultimate authority above these.    **Study supervision (monitoring, data management, etc):** Contract research organization (Clinact)  **Data management team:** Project Manager, eTools Project Manager, Data Manager and Lead Data Manager Reviewer from the Contract research organization (Clinact); Sponsor representatives | |
|  | **Data safety monitoring board (DSMB):**  *Jean-Baptiste Lascarrou - CHU de Nantes (Nantes, France)*  *Guillaume Debaty - SAMU de l'Isère, CHU de l'Université Grenoble Alpes (Grenoble, France)*  *Hans Friberg - Skånes universitetssjukhus, Centrum för hjärtstopp, Lunds universitet (Lund, Sweden)*  *Janneke Horn - Intensive Care, Amsterdam Neuroscience, Amsterdam UMC (Amsterdam, Netherlands)*  *Oriol Roca - Servei de Medicina Intensiva, Parc Taulí Hospital* *Universitari, Universitat Autònoma de Barcelona (Barcelona, Spain)*    **Role of DSMB:** The DSMB will meet after the enrolment of the first, 4th, 12th and 24th patient. In case of any lethal event during the Vent2Cool procedure (i.e., during the connection of the patient ETT to Vent2Cool system), the DSMB will also meet. The DSMB will provide some advice to the Sponsor on pursuing the inclusion or requesting modifications or interruption in patient’s enrolment, after having reviewed safety records and reported adverse events. | |
| **Introduction: Participants, interventions, and outcomes (6 to 8)** | | |
| Described in the main manuscript: Background and rationale, Objectives, Trial design | | |
| **Methods: Participants, interventions, and outcomes (9 to 15)** | | |
| Described in the main manuscript: Study setting, Eligibility criteria, Interventions, Outcomes, Participant timeline, Sample size, Recruitment | | |
| **Methods: Assignment of interventions (16 and 17)** | | |
| Not applicable (Single arm study) | | |
| **Methods: Data collection, management, and analysis** | | |
| **Data collection methods (18a)** | | Source documents for this study will include hospital records and procedure reports and data collection forms. These documents will be used to enter data on the e-CRFs. Data reported on the e-CRF that are derived from source documents must be consistent with the source documents or the discrepancies must be explained.  All data will be collected and entered directly into the Electronic Data Capture (EDC) system in a pseudonymised way. All participating sites will have access to the data entered regarding the individual site’s own enrolled patients. All sites will be fully trained on using the on-line EDC, including eCRF completion guidelines. Investigators and data entry staff will be able to access their account with a username and password. All eCRFs should be completed by designated, trained personnel or by the study coordinator, as appropriate. In all cases, the eCRF should be reviewed, electronically signed, and dated by the Principal Investigator once all data for a specific patient are entered and validated. All changes or corrections to eCRFs will be documented in an audit trail and an adequate explanation for such changes or corrections will be required.  Visual and/or electronic data review will be performed to identify possible data discrepancies. Manual and/or automatic queries will be created in the EDC system and will be issued to the site for appropriate response. Site staff will be responsible for resolving all queries in the database. |
| **Data collection methods (18b)** | | In case of patient’s death before being able to provide a consent or not, if, despite all efforts to contact trusted person or family member, no one could be reached until the end of the study, data will be included in the analysis.  In case of a patient discharged form hospital and lost in follow-up before the end of the study, the patient will be replaced but all data concerning this patient will be analysed until the last available visit, except if an objection to the consent was expressed by the patient, trusted person or family member. |
| **Data management (19)**  Handling of data and data protection | | Eligible patients will be enrolled in the study and sequentially assigned an identification number. This number will begin with one digit representing the site (e.g., ‘1’ for ‘France’ and 2 for ‘Belgium’), followed by a two-digit patient identifier starting with ‘01’. The patient identifier will be assigned to each patient at a site sequentially. For example, the third patient number for site 1 will be 1-03.  In this study, the Contract Research Organization (CRO) processes personal data of patients on behalf of the Sponsor, in accordance with the rules on the protection of personal data and, in particular, the European General Data Protection Regulation (GDPR) EU 2016/679 and of the Council of 27 April 2016 on the protection of natural persons with regard to the processing of personal data and on the free movement of such data. For this purpose, the CRO limits the collection and use of personal data to that which is needed for analysis and control purposes, by ensuring their security and integrity and by guaranteeing their confidentiality.  The CRO makes sure beforehand and throughout the duration of the data-processing:  - of the compliance with the obligations of the applicable data protection law,  - to inform patients of their personal data-processing and obtain their consent on it,  - to implement and maintain appropriate technical and organizational measures.  According to Article 14 of the GDPR, the concerned patient must be informed of the identity and the contact details of the Controller and, where applicable, of the controller's representative.  In France, this research is governed by the CNIL (French Data Protection Agency) “Reference Methodology for processing personal data used within the scope of health research” (MR001). Orixha, as sponsor of the research, has signed a declaration of compliance with this “Reference Methodology”. |
| **Data management (19)**  Data archiving | | The Investigator will maintain, on site, in original format, all essential study documents and source documentation that support the data collected on the study patients in compliance with ISO 14155 and Good Clinical Practice (GCP) guidelines. Documents must be retained for at least 15 years after the end of the study or its completion. These documents may be retained for a different period by agreement with the sponsor or in compliance with pertinent individual country laws and regulations.  The Investigator must consult a representative of the sponsor before disposal of any study records and must notify the sponsor of any change in the location of the study files.  The Sponsor has the responsibility of archiving the CIP, documentation, approvals, and all other essential documents related to the study, including certificates that satisfactory audit and inspection procedures have been carried out if applicable.  These activities will be delegated to the CRO that will archive all documents concerning the study as detailed below:  - All documents must be archived in a secure place and treated as confidential material.  - Paper documents relating to this study will be stored for a maximal duration of one year after the end of the study on site before transmission for archiving to an approved service provider.  - Data will be archived securely as digital and paper version for 15 years from the date of publication of the final report’s acceptance. |
| **Data management (19)**  Final disposition and future use of samples obtained from patients | | A bank of blood samples will be stored at -80°C In the biobank platform of Henri Mondor Hospital (Plateforme de Ressources Biologiques, Hôpital Henri Mondor, 1 rue Gustave Eiffel, 94000 CRETEIL, FRANCE) for 10 years. They will be used for potential ancillary studies studying the effect of Vent2Cool on inflammatory (cytokines) and kidney injury markers in the blood, as well as marker of, in future studies.  Samples will be identified by the study acronym, a pseudonymized code to identify the patient and no personal data from the patient (name, date of birth…) shall be printed on samples tubes. |
| **Statistical methods (20a and 20b)** | | Data analyses will be performed using SAS Version 9.4 or further (SAS Institute, Cary, NC USA software).  Data will be expressed in a descriptive manner using either percentage, when applicable, or both median ± IQR and mean ± SD for other parameters. Confidence intervals at 95% will be calculated according to Wilson’s method.  The primary criteria is the success of the procedure. Our hypothesis is to demonstrate a theoretical success rate greater than 59% with a risk of error of less than 5%.  The success of the procedure will be defined as the ability to reach a body core temperature (bladder temperature recommended) of 33.0±0.5°C and a safe return to conventional mechanical ventilation within less than one hour after Vent2Cool start. Start of the Vent2Cool procedure is defined as the opening of the Vent2Cool patient connector valve.  Based on an estimated success rate of around 80%, a sample of 24 analyzable patients was obtained using Wilson's score interval method for small samples, to achieve sufficient precision of the success rate, with a 95% confidence interval. In fact, this sample will enable us to have a 95% confidence interval whose lower bound will be almost > 60%. Specifically, 19 of the 24 patients will have to undergo the procedure successfully, giving a success rate of 79.17%, Wilson's IC95 [59.53 ;90.76] %.  The mRS, Glasgow score (Glasgow coma scale), Glasgow outcome score, CAHP score will be evaluated in the secondary endpoints. We will calculate the number of patients for which the CAHP score predicted a poor outcome demonstrating an ultimate good outcome determined by mRS <3 or mRS <4 at day 28. Conversely, we will calculate the number of patients for which the CAHP score predicted a good outcome with an ultimate poor outcome determined by mRS ≥3 or mRS ≥4 at day 28. |
| **Statistical methods (20c)**  Missing data | No strategy for the handling of missing data has been defined. Data that are not valid or missing will be considered and treated as missing data. | |

| **Methods: Monitoring** | |
| --- | --- |
| **Data monitoring (21 a)** | Monitoring will be performed during the study according to the Monitoring Plan to assess continued compliance with the protocol and applicable regulations. In addition, the monitor verifies that study records are adequately maintained, that data are reported in the electronic Case Report Form (e-CRF) in a satisfactory manner with respect to timeliness, adequacy, and accuracy, and that the Investigator continues to have sufficient staff and facilities to conduct the study safely and effectively. Further details are described in the Monitoring Plan. The Investigator/institution guarantees direct access to original source documents by the Sponsor, their designees, and appropriate regulatory authorities. The study may also be patient to a quality assurance audit by the Sponsor or its designees, as well as inspection by appropriate regulatory authorities. It is important that the Investigator and relevant study personnel are available during on-site monitoring visits or audits and that sufficient time is devoted to the process.  All monitoring activities will be conducted by the Contract Research Organization CLINACT. The monitoring activity will be carried out throughout the period of subject inclusion and follow-up. The recruitment period is expected to last 11 months, during which the sites will need to recruit 24 subjects. A laboratory manual outlines the activities for collecting, storing, and analyzing the samples. A main laboratory manual details the sample collection at the site and the management of the samples.  During the trial, each Clinical Research Assistant (CRA) may be accompanied by the CLINACT Project Manager (or the delegated person) for a co-monitoring visit to ensure the quality of the monitoring." |
| **Data monitoring (21b)**  Interim analysis | An interim analysis of the data is planned after the 12^th^ patient. Based on the result of the interim analysis, and considering DSMB opinion, the promoter could decide to stop the study. The main criteria will not be calculated during this Interim analysis, so no adjustment of alpha risk of error will be necessary. |
| **Harms (22)**  Recording of AEs | All adverse event (AE), serious adverse event (SAE) and device deficiency (DD) must be collected in detail in the eCRF by the investigator.  The investigator will record all reported events with start dates occurring any time after informed consent is obtained until the last day of study participation.  All AEs shall be documented, in a timely manner throughout the clinical investigation, in the “adverse event documentation” form. Investigators are responsible for documenting AEs.  All AEs will be followed to adequate resolution (resolution or stabilization at a level considered acceptable by the investigator or return to the previous state) even if the patient has terminated his participation in the trial.  This information will be reported at the end of the study in the clinical report. |
| **Harms (22)**  Reporting of SAEs and DD | The Investigator shall report to the sponsor all of the following:   1. any adverse event of a type identified in the clinical investigation plan as being critical to the evaluation of the results of that clinical investigation; 2. any serious adverse event; 3. any device deficiency that might have led to a serious adverse event if appropriate action had not been taken, intervention had not occurred, or circumstances had been less fortunate; 4. any new findings in relation to any event referred to in points (a) to (c).   All these reportable events require reporting by the Investigator to the sponsor immediately, but not later than 3 calendar days after investigation site study personnel’s awareness of the event.  The investigator must document the SAE or DD and evaluate duration, severity, seriousness, causal relationship between the serious adverse events and the procedures added by the research. The action taken and the outcome must also be recorded.  Whenever possible, the investigator will provide the sponsor with any documents that may be useful (medical reports, laboratory test results, results of additional exams, etc.). These documents must be anonymized.  Notification of reportable events must be provided in a written report using the special form for reporting (SAE form / DD form). The report must be completed in the eCRF, validated, printed, and signed by the investigator.  Completed and signed SAE or Device deficiency report (initial notification or follow-up) must be sent to the sponsor and by delegation to the Contract Research Organization (CLINACT's vigilance department).  All SAE considered to be due to the medical device or to the investigational procedures must be reported indefinitely (no time limit).  The initial report sent to the sponsor (by delegation CLINACT 's vigilance department) must be rapidly followed up by one or more additional written reports describing the course of the event and any complementary information.  Whenever possible, the investigator will provide the sponsor (by delegation CLINACT 's vigilance department) with any documents that may be useful for medical assessment of the case (medical reports, laboratory test results, results of additional exams, etc.). These documents must be anonymized. |
| **Hams (22)**  Sponsor responsibilities | The sponsor, represented by CLINACT' s vigilance department continuously assesses the safety of the patients throughout the research. The reception of SAE/DD report form to CLINACT's vigilance department is centralized to allow immediate processing of SAE/DD as soon as they are received.  For any SAE or DD received, the sponsor shall assess:   1. the seriousness of all events reported, 2. the causal relationship between these adverse events and investigational device and/or investigational procedure. By default, events occurring during the Vent2Cool procedure (i.e. when the patient is connected to Vent2Cool) or during the hour following the end of the procedure will be considered as device/procedure-related. Events occurring after this delay will be specifically evaluated for potential causal relationship. 3. the expectedness of the serious adverse reactions based on the information described in the brochure investigator (BI).   Any serious adverse reaction whose nature, severity, frequency, or outcome is inconsistent with the safety information described in the investigator's brochure or in the CIP, will be considered unexpected.  The sponsor (by delegation CLINACT's vigilance) is responsible for declaring to all to National Competent Authorities where the clinical investigation is authorized to start, all of the following:   1. any serious adverse event that has a causal relationship with the investigational device, the comparator or the investigation procedure or where such causal relationship is reasonably possible; 2. any device deficiency that might have led to a serious adverse event if appropriate action had not been taken, intervention had not occurred, or circumstances had been less fortunate; 3. any new findings in relation to any event referred to in points a) and b)   Reportable events must be reported at the same time to all NCAs using the table "Investigation summary safety report form" (MDCG-2020-10/2 rev1):   1. For all reportable events which indicate an imminent risk of death, serious injury, or serious illness and that requires prompt remedial action for other patients, users or other persons or a new finding to it: Immediately, but not later than 2 calendar days after awareness by sponsor of a new reportable event or of new information in relation with an already reported event. 2. Any other reportable events as described in section 5 or a new finding/update to it: Immediately, but not later than 7 calendar days following the date of awareness by the sponsor of the new reportable event or of new information in relation with an already reported event.   The table gives a cumulative overview of the reportable events per clinical investigation and will be updated and transmitted to participating NCAs each time a new reportable event or a new finding to an already reported event is to be reported. If necessary, more detailed information will be provided at the request of the NCAs.  The sponsor (by delegation the CLINACT's vigilance) must notify all the investigators involved about any information that could adversely affect the safety of the research participants. |
| **Auditing (23)** | A minimum of 9 monitoring visits by CLINACT is planned at each center during the study, to ensure a visit for every 2 inclusions. The first monitoring visit will be organized within 2 weeks following the inclusion of the first patient at both centers, and the second visit after the follow-up period of the first patient in the study. The final monitoring visit will be organized within 2 weeks following the last visit of the last patient at each center. |

| **Ethics and dissemination** | |
| --- | --- |
| **Research ethics approval (24)** | The study will be performed in accordance with the following applicable standards and regulations:  - EN ISO 14155:2020 on clinical investigations with medical devices on human patients  - recommendations guiding physicians in biomedical research involving human patients adopted by the 18th World Medical Assembly, Helsinki, Finland, 1964 and later revisions  - EU Medical Device Regulation 2017-745  - applicable national regulations  It is the Sponsor’s responsibility to ensure the CIP, informed consent, any other specific study documents and all amendments to these study documents are reviewed and approved by the appropriate Ethics Committees and Competent Authority before enrolment of any patient. The regulatory activities will be delegated to the CRO. |
| **Protocol amendments (25)** | The Sponsor or the Investigator might modify the protocol for ethical, medical or scientific reasons.  No change can be implemented at investigational sites, unless to eliminate an immediate hazard to study patients, without having obtained the favourable opinion of the Sponsor.  These changes will be documented in the Trial Master File (TMF) and communicated to investigational centres as soon as possible.  Any changes to the protocol will be submitted as substantial or non-substantial modification (according to its nature) to the responsible ethics committee and/or the national health competent authority. |
| **Consent (26a)**  Who will obtain informed consent or assent from potential trial participants or authorised surrogates, and how | In the context of comatose patients resuscitated after cardiac arrest, it will not be possible to obtain prior and informed consent from the patient himself.  The investigator will provide a written information note and orally explain the study to the family member or trusted person and the written consent of the family member or trusted person will be obtained. The patient's confirmation of consent will be systematically sought as soon as possible.  In the event of the absence of a family member or trusted person at the time of inclusion, inclusion will be possible, and patient will be included via an emergency inclusion procedure. The patient's confirmation of consent will be systematically sought as soon as possible. The signature of the consent form will therefore not be systematically required for inclusion.  Confirmation of consent from surviving patients will be obtained as soon as they become able to receive all the information necessary to give their consent. The search for consent to pursue will continue until Day 28, the date of the last day of participation in the study. If on this date the patient is still unable to give his consent, a request for written non-objection will be made to the trusted person or a family member and recorded in the patient's medical file. In case of non-objection, the data collected from this patient will be analysed.  A request for written non-objection will also be addressed to the trusted person or a family member in the event of the patient's death during his follow-up and before obtaining the consent to prosecute. In case of non-objection, within a reasonable delay (1 month), the data of this patient will be analysed.  In case of patient’s death before being able to provide a consent or not, if, despite all efforts to contact trusted person or family member, no one could be reached until the end of the study, data will be included in the analysis.  All of the steps taken by the investigator and the responses of the trusted person or a family member must be explicitly noted in the patient's medical file. |
| **Consent (26b)**  Additional consent provisions for collection and use of participant data and biological specimens in ancillary studies | Patient will be informed during the inclusion and consent process of the possibility to participate in a future observational study allowing follow-up after Day 28.  In the initial consent process, blood samples collection and storing is planned in a central biobank (Mondor Hospital, Creteil, France) for the further evaluation of Kidney Injury Molecule (e.g., KIM1; renal dysfunction marker), blood cytokines (e.g., Interleukin-6) and total blood transcripts. The results of these analyses are not included in the initial data management plan. |
| **Confidentiality (27)** | All documents will be stored safely in confidential conditions. On all study-specific documents other than the signed consent, the patient will be referred to by the study patient identification number. |
| **Declaration of interests (28)** | The coordinated investigator (Alain Cariou) does not perceive any honorarium from the study sponsor.  The scientific supervisor (Renaud Tissier) is acting medical advisor of the Sponsor. He is also a cofoundor of Orixha (Sponsor) and perceives honorarium from the company.  Other members of the Steering committee and DSMB could perceive honorarium from the sponsor. |
| **Access to data (29)** | Statement of who will have access to the final trial dataset, and disclosure of contractual agreements that limit such access for investigators |
| **Ancillary and post-trial care (30)** | Patient will be informed during the inclusion and consent process of the possibility to participate in a future observational study allowing follow-up after Day 28. This observational study will follow all patients on whom the Vent2Cool procedure has been used, starting with the OverCool study and including all future interventional studies.  Patients will be contacted by the investigators once a year for five years to obtain a long-term view of patients’ outcomes after the Vent2Cool procedure.  This observational study is currently being prepared and will be submitted to the appropriate regulatory bodies 4 months after the first inclusion in OverCool study, at the latest, to ensure first inclusion in the observational study will be possible not later than 1 year after the last visit of the first patient included in OverCool study.  In addition, potential ancillary studies studying the effect of Vent2Cool on inflammatory (cytokines) and kidney injury markers in the blood could be proposed using the patients’ blood samples stored in a Biobank. The patient consent form includes this mention. |
| **Dissemination policy (31a, 31b, 31c)** | The Clinical investigation design will be registered in a publicly accessible database.  The protocol summary will also be published in a scientific journal. The authors will the principal investigators the scientific coordinator and the others member of the Scientific Committee of the study  At the end of the Study, the results will be analysed in a Statistical Report, validated by the Sponsor. The final Statistical Report will be signed by the Biostatistician and the Sponsor.  Results will then be published as a Clinical Investigation Report, based on a Statistical Report, in compliance with EU MDR 2017-745 and ISO 14155:2012 will be after Sponsor validation. This final report will be signed by the principal investigator, the Author of the report, and the Sponsor.  The results of the clinical investigation will also be published in a scientific journal. Authors will be investigators and members of the steering committee. The manuscript is planned to be drafted by the scientific supervisor and coordinating investigator. |
| **Appendices** | |
| **Informed consent materials (32)** | Consent forms can be provided on request to the corresponding author. |
| **Biological specimens (33)** | A bank of blood samples will be stored at -80°C In the biobank platform of Henri Mondor Hospital (Plateforme de Ressources Biologiques, Hôpital Henri Mondor, 1 rue Gustave Eiffel, 94000 CRETEIL, FRANCE) for 10 years. They will be used for potential ancillary studies studying the effect of Vent2Cool on biological markers in the blood in future studies.  Samples will be identified by the study acronym, a pseudonymized code to identify the patient and no personal data from the patient (name, date of birth…) shall be printed on samples tubes. |
